# Public Claims about Automatic External Defibrillators: An Online Consumer Opinions Study

**DOI:** 10.1186/1471-2458-11-332

**Published:** 2011-05-18

**Authors:** Arthur G Money, Julie Barnett, Jasna Kuljis

**Affiliations:** 1 Department of Computer Science and Technology, University of Bedfordshire, Luton, Bedfordshire, LU1 3JU, UK; 2 Department of Information Systems and Computing, Brunel University, Uxbridge, UB8 3PH, UK

## Abstract

**Background:**

Patients are no longer passive recipients of health care, and increasingly engage in health communications outside of the traditional patient and health care professional relationship. As a result, patient opinions and health related judgements are now being informed by a wide range of social, media, and online information sources. Government initiatives recognise self-delivery of health care as a valuable means of responding to the anticipated increased global demand for health resources. Automated External Defibrillators (AEDs), designed for the treatment of Sudden Cardiac Arrest (SCA), have recently become available for 'over the counter' purchase with no need for a prescription. This paper explores the claims and argumentation of lay persons and health care practitioners and professionals relating to these, and how these may impact on the acceptance, adoption and use of these devices within the home context.

**Methods:**

We carry out a thematic content analysis of a novel form of Internet-based data: online consumer opinions of AED devices posted on Amazon.com, the world's largest online retailer. A total of #83 online consumer reviews of home AEDs are analysed. The analysis is both inductive, identifying themes that emerged from the data, exploring the parameters of public debate relating to these devices, and also driven by theory, centring around the parameters that may impact upon the acceptance, adoption and use of these devices within the home as indicated by the Technology Acceptance Model (TAM).

**Results:**

Five high-level themes around which arguments for and against the adoption of home AEDs are identified and considered in the context of TAM. These include opinions relating to device usability, usefulness, cost, emotional implications of device ownership, and individual patient risk status. Emotional implications associated with AED acceptance, adoption and use emerged as a notable factor that is not currently reflected within the existing TAM.

**Conclusions:**

The value, credibility and implications of the findings of this study are considered within the context of existing AED research, and related to technology acceptance theory. From a methodological perspective, this study demonstrates the potential value of online consumer reviews as a novel data source for exploring the parameters of public debate relating to emerging health care technologies.

## Background

The nature of health communication that occurs between patients and health care professionals is changing. Now more than ever, patients engage with sources outside of the traditional patient/health professional relationship [1]. As a consequence, patients are taking personal responsibility for their own health care by making their own health related decisions and judgements [2]. With the advent of the Internet, patients and carers are now also accessing online discussion forums and self-help groups which may serve as valuable sources of health care support and information [3]. In many cases, health care professionals welcome and encourage this shift [4], recognising the associated health care benefits that expert patient knowledge and support can provide. The involvement of both patients and, where appropriate, carers, is seen to ease of burden that would otherwise be placed on traditional health resources [5]. For others, questions still remain over the provenance and reliability of these external sources, particularly in the case of information sourced from the Internet [6]. However, the ubiquity, if not the potential value of alternative sources is becoming increasingly recognised. Arguably to some extent health communication has come full-circle, with expert patients now serving as valuable sources for consultation in the education of health care students and experts [7,8].

With an anticipated rise in demand for health care resources as a result of an ageing population [9,10], government initiatives see patient participation in the delivery of health care as one of the few areas in which there still remains capacity for reducing costs and improving quality of service provision [11,12]. As well as the availability of an increasing range of information sources, a further area that exemplifies this trend is the rise in direct-to-consumer self-treatment devices. These are increasingly being made available 'over-the-counter', both on the high-street and online. Patients are now able to make private purchases of a range of health care devices that enable self-diagnosis, self-monitoring and self-treatment. By virtue of this, both carers and the 'cared-for' [13] are becoming more equal partners in health care provision alongside health care professionals, and in some cases, relegating or even excluding health care providers from the health care decision making process [14].

With rising numbers of the population having access to the Internet, patients more frequently use the Web as a first port of call when seeking out health information, advice, and support [15,16]. Not only can patients access health information via official online sources, such as NHS direct [17], the interactive nature of the Internet allows patients to become active participants in patient-led online discussion forums, consumer opinion sites, mailing lists, and blogs [18]. These sites provide a virtually limitless source of highly personalised support and advice, based on first-hand experience with illness which can be easily accessed at any time [19]. Such sites are of significant value allowing patients to make connections and share critical evidence-based information relating to their individual conditions [20]. Furthermore, they can provide a source of emotional support, and may also assist the patient in making informed decisions about their own health care, and hence to feel more in control of their own life and health [21] which has been linked to improved levels of compliance with treatment [22,23].

One location where consumer views relating specifically to self-care devices, as well as health issues in general are re-presented is online consumer opinion forums. It is to the potential value, and the validity of using these as a source of data, that we now turn.

### Online Consumer Opinion Forums as a Source of Data

Online consumer opinion forums adopt a similar interaction model to that employed by Web-based discussion threads/forums (sometimes referred to as weblogs), allowing users to express opinions relating to a specific product, against a backdrop of other consumer opinions that have been previously posted relating to that product. The key factors that differentiate weblogs from consumer opinion forums are that, in the case of the latter, user comments are initiated around the product for which the consumer opinion forum is dedicated, whereas in the case of the former discussion may be initiated around any topic, not necessarily product related.

There are a number of characteristics of consumer opinions fora that should be borne in mind where these are being used for data. First, this represents an arena where it is expected that people will be clear in communicating the key plus and minus points of a product, and provide a resource for other people interested in such products to use as a means of informing their decision making [24]. In that sense they are public utterances in a rather different way than are the opinions expressed in the more constrained arena of an interview or focus group. Second, the comparative anonymity that the Internet affords users, may allow them to share opinions that they may not otherwise debate in more traditional face-to-face research settings [25,26]. The Internet also provides the opportunity to gain insights from users that may not be reached via more traditional participant recruitment methods [27]. Arguably online consumer opinion data is a potentially useful data source: it reflects, at least to some extent, consumer views about a particular product and yet at the same time may contribute towards forming the opinions of other consumers. There is certainly increasing evidence that electronic word-of-mouth has a significant influence on public opinion and purchasing behaviour [28]. For example, a survey of Bizrate.com found that 44% of users consulted consumer opinion sites prior to making a purchase [29]. This survey also found that 59% of respondents considered consumer-generated reviews to be more valuable than expert reviews.

Turning to potential drawbacks of this type of data, as the researcher may not have the opportunity to ask follow up questions, it is sensible to ensure that the data is sufficiently rich in content prior to embarking on time consuming analysis [30]. In terms of representative samples, the demographics of contributors may be skewed towards representing those users that have access to the Internet or those that have a need to express an opinion about a given product [31]. Indeed it is feasible that those individuals that do contribute to such fora may not be at all similar to those that do not contribute or have the opportunity to do so. Nevertheless, they do provide a readily available and naturally occurring means of gaining insights into the views of a sub-set of consumers that otherwise may be difficult to achieve. Little is known about the integrity of the data, or indeed to what extent it truly reflects user's real-life personal views. It is possible that the content of users' contributions is often made deliberately controversial, in an attempt to encourage others to make their opinions heard, whilst others may just like the idea of seeing their own opinions in print [32]. However, other more conventional research methods are similarly subject to unseen agendas of participants. In support of the integrity of discussion thread data, Hennig-Thurau [24] found that a key factor motivating users to contribute to consumer opinion forums, is a genuine concern for the wellbeing of other consumers. Whatever the motivation, in reality the opinions that are expressed on consumer opinion forums may be considered relevant, because this is what users browsing the forum can comprehend from this content [32].

### Automated External Defibrillators: Changing Context of Use

Sudden Cardiac Arrest (SCA) is the leading cause of death across the world, claiming up to 400,000 lives in the United States [33,34] and over 100,000 lives in the United Kingdom every year [35]. An Automated External Defibrillator (AED) is a device used, traditionally by trained health professionals, in the treatment of acute incidents of SCA. Typically, the AED is used to re-establish regular heart rhythm by placing adhesive pads on the patient's chest and delivering an electric shock to the heart when the heartbeat appears to be dangerously fast as a result of a ventricular tachycardia or ventricular fibrillation [36]. Survival rates for SCA are primarily determined by the length of time that elapses between the onset of the SCA episode and administering electrical defibrillation, i.e. the faster the intervention, the higher the survival rate [37]. As a result, there have been a number of initiatives to make AEDs more widely available in public places such as train stations, airports, shopping centres and ferry ports [38]. To date over 6000 defibrillators have been placed in the community via a scheme managed by the British Heart Foundation (BHF) [39].

Although the placement of AEDs in public places continues to be seen as a valuable initiative, statistics show that approximately three quarters of SCA deaths do not occur in public places, but rather within the home [34]. As a result, more recently, manufacturers have started to focus their attention on developing AEDs for use within the home by untrained lay care-givers and members of the public. Manufacturers claim that home AED devices are designed for 'extreme ease of use by laypeople' [40]. Home AEDs provide users with detailed and step by step real-time spoken instructions and visual aids, supporting the user to administer the device effectively in the event of a SCA emergency. The AED guides the user through the stages of administering Cardiopulmonary Resuscitation (CPR), placing of the adhesive pads on the chest of the patient, and suggests contacting emergency services if possible. It then monitors the patient's heart rate and diagnoses and administers a shock automatically if appropriate.

Empirical research, comparing the effectiveness of home AEDs used by untrained laypersons with trained laypersons, concluded that home AEDs can be effectively used by untrained laypersons, and that time-consuming AED training programmes may not be necessary [41]. Similar conclusions were drawn in a two year study carried out in O'Hare Airport in Chicago [42], which placed 33 public access AEDs intended for use by untrained bystanders. However, despite the relative ease of use of the device, when witnessing an SCA event within the home with an AED at hand, a significant number of trained family members failed to even attempt to attach the AED pads to the patient [34]. There is a need to better understand the emotional and psychological implications that individuals perceive to be associated with making the decision of whether to deploy the device in these emergency situations.

Little research has been carried out to explore patients' attitudes towards home AED's and the factors that affect their acceptance and use within the home setting. McDaniel, Berry, Haines, and DiMarco [43], in a quantitative study, explored the practical and psychological aspects of training high risk SCA patients and their significant others in the use of home AEDs. The study found no unfavourable psychological impact of training. Inspired by this study, Chen, Eisenberg and Meischke [44] conducted a qualitative study to gain insights into the perceptions of high risk SCA patients and their significant others of having AEDs placed within the home. The results revealed that patients generally noted only positive effects of having AEDs within the home, and that significant others had high levels of confidence in their ability to appropriately use the home AED unit.

### Technology Acceptance, Adoption and Use

Over the past two decades, much research effort has been invested into understanding user end users' reactions and motivations to technology acceptance, adoption and use [45]. There is increasing interest in gaining a better understanding of the factors that influence user acceptance, adoption and use of technology within the health care domain [46]. To the best of our knowledge, there is no research yet that specifically explores barriers to acceptance, adoption and use of medical devices within the home. The Technology Acceptance Model (TAM) is perhaps the most notable theory applied in the explanation of user motivations, attitudes and responses to acceptance and use of technology [47]. Despite its relative simplicity, the most basic form of TAM is typically seen to provide an explanation of approximately 40% of issues related to technology acceptance [45].

TAM proposes that when presented with a new technology, users' Behavioural Intention to use (BI) and their Actual Use (AU) of technology are typically mediated by two key factors: Perceived Usefulness (PU) - the extent to which the user perceives that the new technology will aid them in performing the task at hand, and Perceived Ease of Use (PEOU) - the extent to which the individual believes using the technology would be free of effort [48].

TAM is now increasingly being applied within the health care research domain [49]. Some examples include exploring the acceptance of: Personal Digital Assistants (PDAs) by physicians [50]; and a range of customisable and wearable health care devices for patients and practitioners [51]; portable postural assessment technologies for use by physiotherapists [52]; mobile picture archiving technologies for dental care [53]. However it has been noted that the generic TAM model may often not be appropriate for considering acceptance of health care technologies [50,54] and that a number of modifications and additions are necessary for it to be optimally applied to technologies within a health care context [54-56].

Thus far no research has been carried out to explore the extent to which the generic TAM model predicts user acceptance of AEDs or indeed whether additional features may influence their acceptance, adoption and use. It is likely that there are device specific acceptance factors, given that AEDs are health care devices, but perhaps even more so since they are used within the emergency context, which was found to have a considerable impact on the applicability of TAM [57]. Furthermore, no qualitative research to date appears to exploit any form of naturally occurring Web-based data as a potential means of exploring and scoping the applicability of TAM.

As a first step towards gaining a better understanding of public attitudes towards home Automated External Defibrillators (AEDs), the factors that affect their acceptance, adoption and use within the home context, as well as the potential value of online consumer opinions as a data source, we have explored opinions of home AED devices posted on Amazon.com. The objectives of this study are three-fold and aim to make some progress in answering the following questions:

• What are the valued properties of this medical device and what factors appear to influence their acceptance, adoption and use?

• How do the findings of this study compare with similar research that has sourced primary data via more traditional methods, such as semi-structured interviews?

• Does online consumer opinions data serve as a potentially valuable naturally occurring Web-based data source for exploring and scoping the applicability of TAM to a specific technology?

## Methods

### Data Collection

Amazon.com is the world's largest online retailer and also hosts one of the largest online consumer opinion services. It allows users to post consumer opinions about any products that are sold on this online retailing site. Site users are asked to rate the product with one to five stars and to provide a textual description of their opinions about that product. Similar to weblogs, users are free to comment on previously posted comments. Amazon.com does not pay users for making posts. Any user that has an opinion is free to comment, as users do not have to have purchased the product in order to contribute. Furthermore, in the interests of equality and clarity of users' views, Amazon.com forums only allow users to make one post per product listed on the site.

For the purposes of this study, we collected all consumer opinions posted on Amazon.com relating to AEDs sold for use within the home. This consisted of a total of 83 posts, representing 83 separate user's opinions on two leading brands of home AEDs. Posts were made on the site between October 2004 and September 2009. The average length of each post was 214 words, each post varying in length between 26 to 998 words.

### Data analysis

After identifying AED consumer opinions, the data was collated into one document. Each individual participant's contribution to the discussion thread was allocated a unique identifier, for reference purposes, a number between #1 and #83, #1 being the most recently posted opinion and #83 being the oldest posted opinion.

Established thematic analysis techniques were used to analyse the data [58]. The aim of thematic analysis is to "describe how thematic contents are elaborated by groups of participants, and to identify meanings that are valid across many participants" [59]. The approach taken to this was both inductive, as some themes were closely linked to the data, and other themes were driven by theory and the researchers' analytical interest [60]. In the case of the latter, researchers' analytical interests were driven by issues relating to TAM, and hence focused on the factors that appeared to influence the acceptance, adoption and use of home AEDs.

The entire dataset was closely read and patterns in the data were noted. Sequences of data that represented "the most basic segment, or element, of the raw data or information that can be assessed in a meaningful way regarding the phenomenon" [58] were identified and assigned a code name. The dataset was then examined iteratively through several stages of splicing, linking, deleting and reassigning themes and sub-themes. The first and second authors coded the data and discussed inconsistencies where these arose until a clear consensus of the main themes was reached. The main themes are those drawn from multiple contributions and that represent issues that are clearly central to the participants themselves. Within these themes we have identified sub-themes that depict the breadth of positions that were adopted within the main themes.

## Results

Before exploring the main themes that were evident in the postings that were made about AEDs, it is useful to reflect on the self-attributions made by the participants as these are often of use to warrant the claims being made and can thus also be used to interpret the breadth of positions that are adopted on particular issues.

Out of 83 posts, 47 individuals argued strongly for AEDs, 26 individuals argued for AEDs but with caveats, eight argued against AEDs and two were impartial. Twenty two individuals/posts explicitly claimed to own AEDs, 15 made no claims to owning an AED and 46 made no claims about AED ownership. A total of 37 individuals stated that they had some level of medical expertise, these ranged from having attended basic first aid courses to being fully qualified doctors. Forty-six individuals made no claims to having medical expertise. Quotes presented in this section originating from individuals claiming some level of medical expertise are appended with "MED", and those that made no claims of medical expertise are appended with "NON-MED".

The parameters of dialogue between lay people and health care practitioners and professionals concerning home AEDs emerged via five high-level themes: Usability: physical device design features of the AED that affect its efficient use; Usefulness: the extent to which AEDs are perceived as providing an effective treatment for SCA events; Cost: the cost of purchasing the AED; Emotional implications: the emotions invoked by AED purchase or ownership; Risk status: the relationship between the perceived level of SCA risk and AED purchase or ownership.

As a measure of consistency, 25% of the data (21 of the 83 posts) was independently coded according to the main themes agreed by the first and second authors, so that inter-rater reliability could be calculated. The results revealed with Cohen's Kappa value of 0.819 with a significance of p < .000, which indicates an excellent level of agreement [61]. Table 1 provides a frequency count of the themes as they arose within the 83 posts. Some segments of text were allocated to more than one theme.

**Table 1:** frequency count of consumer opinion themes

Consumer opinion themes	Frequency
Usability	41

Usefulness	51

Cost	26

Emotional implications	21

Risk	28

### Usability

Discussions relating to the usability of AEDs centred around core and peripheral features of the device. Core features relate to the efficient and effective operation of the device, and peripheral features relate to the device when it was not in operation. The majority of comments related to the assistive features, including spoken and pictorial instructions that prompt and coach the user through every step of the defibrillation process. Users claimed that the most valuable feature of the AED was the minimal amount of input required from the user. This was especially important given that the purpose of the device was to save a life, as illustrated in the quote below.

This device is truly amazing. It really, really is as easy to use as a doorbell. If a shock is needed, you push a flashing button after the devices tells you to do so. The (product name) does all the thinking. Its voice is remarkably reassuring. The instructions are simple and clear. <<#74 NON-MED>>

The automated and assistive nature of these devices was thus not only perceived as being of value by minimising the cognitive load passed on to the caregiver in a highly stressful situation, but was also seen as a way of almost completely passing the responsibility of carrying out the procedure correctly onto the device itself.

It's great these are finally available for the public. This is a very easy to use piece of equipment. SO easy, that even someone who's loved one is laying on the floor can still operate it. Turn it on, stick on the patches and the machine does the rest. <<#66 NON-MED>>

One extension of this argument was that the machine was intelligent enough to ensure that the user would not be allowed to harm someone.

It only shocks if after the device performs an analysis, it determines the shock is appropriate for the situation -- so you don't risk hurting the person in distress by using it, as long as you follow the simple directions. <<#21 MED>>

It was also clear that many contributors to the discussion implicitly recognised that until recently, medical expertise was generally required in order to use a defibrillator and thus the claims that they made around the usability of the device highlighted the legitimacy of the lay user. The device was routinely referred to as being 'idiot proof' and suitable for 'even the most unsophisticated person'.

Peripheral factors included the importance of a device having long battery life, durable casing and a design that allowed it to be easy to carry. Water resistance and weight of device were also noted as valid peripheral usability factors. Regardless of positive comments relating to the device, many users expressed hope that they were never put in a position of having to actually use the device.

Very compact with good instructions. Comes with a very durable case and handle which makes it easily portable. I hope I never have to use it! <#12 NON-MED>

### Usefulness

In terms of the extent to which AEDs provided a useful and holistic solution in the treatment of SCA events, a wide range of opinions were presented. These ranged from perceiving the device as the critical element involved in saving a life; suggesting that its usefulness depends on whether it was embedded with other procedures, through to the proposition that other procedures were more effective.

A common opinion was that the AED is a crucial piece of equipment to have in the home that makes the difference between life and death when responding to a SCA event. Some posts suggested that as the patient requires defibrillation much earlier than is possible via the emergency response services, the use of an AED device is the only means of ensuring a life is saved.

Defibrillation is the most crucial link in the Chain of Survival.....Sudden Cardiac Arrest has no age restrictions...young or old, ...the only and definitive treatment for Sudden Cardiac Arrest is to deliver a shock to the heart! <<#50 NON-MED>>

If you or a loved one "drops dead" with a sudden cardiac arrest, this little box can literally be the difference between life and death. When a defibrillator is used within the first couple minutes following cardiac arrest, their effectiveness in restoring a pulse is very good. By the time paramedics or other trained professionals arrive, it is often too late for successful resuscitation - even when bystanders start performing CPR immediately. This is an excellent product produced by a well respected manufacturer. The machine is simple to use and was, in fact, designed to be used by a layperson in critical, stressful emergency situations. <<#47 NON-MED>>

Perhaps the most commonly voiced opinion about usefulness was that although AEDs are useful, they only contribute part of the solution when treating a SCA event. The other required element was expertise in CPR which would require training. In particular, it was believed that rescuers making use of the AED, should as a minimum have some training in the use of the equipment, so that they are able to make rapid use of the equipment when the need arises. Indeed, regardless of whether the AED can do no harm when used, some still considered that equally the device could not do much good without appropriate training.

Of course you can't hurt anyone with it because it won't defib except under the right conditions but you can't help much either without the proper training. <<#28 MED>>

Furthermore, it was believed that training would ensure that rescuers would appreciate the importance of administering CPR prior to defibrillation or at least until the AED is ready to be used.

The AED is a great device. However, it is only as good as the training you need to receive as a user. CPR is what is going to keep oxygen in the person's system and circulating it while someone goes to get the AED or in a worst case scenario, the rhythm is a non-shockable one. CPR is not a difficult skill but it definitely is not one to learn while you are in the middle of an emergency situation, especially on a loved one. <<#59 MED>>

In contrast to the view that AEDs played a useful role in treatment of SCA, a number of opinions were voiced that questioned the usefulness of these devices. Two main arguments were deployed in order to do this. Firstly, some contributors to the discussion argued that individuals should invest effort and resources into preventing the likelihood of a SCA event occurring in the first place.

Something to consider though, is that $1500 would buy you a pretty nice treadmill, a lot of months at a health club, or many boxes of nicotine patches to quit smoking. You will be better served in the long run if you invest your time in PREVENTING cardiac arrest in the first place by cutting your risk factors...exercise and smoking are two of the biggest. <<#45 NON-MED>>

A much more cost-effective way to increase your longevity is simply to alter your diet. <<#22 NON-MED>>

Secondly, others argued that CPR alone was sufficient and was the single most important treatment that could be provided by lay people in the event of a SCA. A more extreme version of this argument was that the deployment of an AED could be harmful because it diverted resources from the most important action necessary to sustain life, that is, to maintain oxygen circulation throughout the body via effective CPR.

Only then can a defibrillator shock the heart into proper pumping action. The most important thing a layman can do is learn CPR. If you can keep OXYGEN going into the lungs and then circulate that oxygen to the brain and heart (PUMPING) you have done the ONLY really important thing. If people use this for a Myocardial Infarction, INSTEAD of IMMEDIATELY starting effective CPR, the TIME (manufacturer name)causes them to waste deprives the brain of oxygen and dooms the patient. <<#41 MED>>

### Cost

Many of the contributions commented on the cost of the device (typically $1,500). Those arguing in favour of AEDs, typically expressed the view that money was not an appropriate currency in which to characterise the benefits of the device. Others recognised expense as a factor but considered AEDs as a worthwhile purchase if affordable, whilst others stated that money is better spent on other things.

Many opinions expressed the notion that the cost of a home AED was a small price to pay, particularly when set against the value of a life, which, it was argued could not have a price associated with it. Moreover, the idea that money has no value in the event of death made the argument more compelling.

If it seems like a lot of money, without [an AED] money may become un-necessary...... So I guess it may come down to the old axiom: It's your money or your life. <<#66 NON-MED>>

In the contribution below the expense of buying an AED is justified by juxtaposing the cost and benefit of the everyday practice of buying coffee with that of saving a life.

I understand other reviewers' perspectives that the investment may not be 'cost effective', and that the likelihood that you would ever use it is very low and so therefore a 'waste of money'. Yes it is expensive, but you cannot really put a price on a saved life - it is priceless...If you can afford a Starbucks coffee every day, for example, then you can certainly afford to buy one of these. <<#21 MED>>

Financial cost was often weighed against tangible day to day benefits, and anticipated negative emotions. In the first quote below, it was noted that having a device placed within the home provides a valued sense of security and appreciation of how precious life is. The second example juxtaposes the notion of 'the day we need it' against 'the day after'.

Given the thought of needing one and not having it--cost really wasn't much of a consideration. Each time I pass it, (we keep ours in the laundry room) I'm reminded just how precious life is. I highly recommend this product and congratulate (manufacturer name) for its pioneering work in this area. <<#74 NON-MED>>

When some things goes wrong, we always think of what would happen if we just had what we needed in time. I think this might be one of those things we will be very happy to have handy the day we need it. We might have no chance to buy it the day after. <<#75 NON-MED>>

Several contributors explicitly set their willingness to pay for the device against their hope of never having to use it. Using the device to save a life was priceless but never having to use it also brought benefits in terms of knowing that you were equipped to do so.

I hope and pray that this is the "worst" investment I have ever made as I hope to never be in a situation where I would have to actually USE the AED -- but if I ever do find myself where a person in my home is suffering from chest pain or becomes unconscious, I would rather have the AED on hand (and know how to use it) and take the chance that it might help them, but know there is a good chance it might not, but at least I know I did everything I could while waiting for the EMT to arrive.... than not have the AED as an option at all. <<#21 MED>>

There was also a recognition that, for individuals that are at higher risk from SCA, the cost may be even more justifiable, perhaps because the likelihood of the device being used for its intended purpose may also be more probable.

It is not indicated for everyone but, for someone who can spend $1500 and have a heart condition that make them prone to sudden death, this device can be a very justifiable spending.<<#40 MED>>

### Emotional implications

Opinions relating to personal device ownership and use were seen as having a range of perceived emotional implications. The three main facets of emotional implications are: the regret and anxiety associated with not having an AED; peace of mind and joy of saving a life; denial of emotional implications.

The construction of scenarios in which an AED was required, but had not been purchased was used to warn against the regret that would be forthcoming. In some cases, personal accounts from individuals that had been in such a situation were shared, and compelling arguments made for purchasing an AED.

I know from personal experience if your gut is telling you to get one and you don't do it...I won't go into all the emotional part of my story ... But about 8 years ago, I thought about getting one of these but never did. Later in June of that year my father who was staying with me had heart failure. I knew CPR and started it right away, so when EMS got here about 12 minutes later they were able to use their defibrillator right away... without success. Had I had this defibrillator then, would it have saved my fathers life? I don't know for sure, but I sure wish I had had it then!!!!!!! So I'll just say IF you hear that inner voice telling you to get one...seriously consider it! <<#3 MED>>

In order to strengthen the threat of regret, SCA was constructed as being no respecter of persons that can strike with no warning. In the following excerpt, this argument was used to make a stronger case for considering ownership of such a device.

When some things goes wrong, we always think of what would happen if we just had what we needed in time. I think this might be one of those things we will be very happy to have handy the day we need it. We might have no chance to buy it the day after. I saw how it works, and it is really very simple to use, with guidance from the beginning to the end of the process. <<#74 NON-MED>>

Ownership of the device was associated with a sense of relief, peace of mind and reduced worry. In the following quote, the purchase of an AED was constructed as transforming worries that an at-risk parent would not live long enough to see grandchildren grow up into improved quality of life and increased peace of mind.

I bought this for my mother for her 60th birthday. She has very high blood pressure and high cholesterol. There was always this worry hanging over our family. An anxiety that paralyzed all of us a little. We all worried that mom wouldn't see her grandchildren grow up. She worried too. Since I gave her this (granted, unusual) gift, I know with certainty that the anxiety level has gone down. We've all watched the video (including Dad) and we all just feel better about my mother's health - because we know that we're prepared to help her. ...It's just nice to have it. It's not morbid at all. We don't feel helpless anymore - and Mom knows that we know what to do if anything happens. Like everyone else, I hope it's never needed. But it was still worth the $$ for the peace of mind that we have. Mom enjoys life a little more, and we are happier. Life is better when you can live it without worrying all the time. <<#11 NON-MED>>

Purchasing an AED was also constructed as individuals doing everything within their power to prepare for a possible SCA event. Indeed, the consequence of having purchased an AED was seen to give the purchaser peace of mind that they had done everything they could to ensure the maximum chance of survival.

Perhaps this is not true for everybody. But for me, in my own case, it is better to have the peace of mind that I try and do everything I possibly can to help aid and care for a person in distress. <<21 MED>>

In some cases, there seemed to be an element of denial of what the emotional implications of using such a device might be, or indeed what may be required of the user in the event of a SCA event. These owners of AEDs implied that they had done all they could by purchasing the device, and they simply hoped the device was easy to use if ever needed.

God willing, I won't ever have to. When I do need to use it, I hope it is easy. If it's too hard, the consequences could be dire. <<#16 NON-MED>>

### Risk status

Participants often expressed varied opinions regarding SCA health risk status, and the extent to which this should be considered when contemplating the purchase of an AED. There was a feeling that even for individuals that are perceived to have a low SCA risk status, it was still necessary to purchase this product, as even the most unlikely of cases have resulted in death from a SCA event, as indicated in the quote below.

Even people who take care of themselves by living a healthy lifestyle, can still have heart attacks......the first guru of running died of one. <#69 NON-MED>

An extension of this notion was the idea that risk status should not be considered the decisive factor when contemplating the purchase of this product. When considering that SCA could strike anyone at any time, it was implied that individuals had a moral obligation to purchase this device if they truly valued and wanted to protect the lives of those close to them.

I'm a 44 year old man, no family history of heart disease, "normal" blood pressure, slightly elevated cholesterol, exercise 3-4 times per week--frankly, I expect my heart will never need to be defibrillated. But then again, who does? I bought mine to protect myself, my family and friends--even my neighbors. <#74 NON-MED>

Perhaps the most common argument suggested that a range of factors determined an individual's SCA risk status, and hence determined the extent to which an AED could be potentially valuable. Participants engaged in debate regarding the extent to which leading an unhealthy lifestyle placed individuals sufficiently at risk to warrant the purchase of the device, or whether a family history of heart disease was the over-riding factor in determining risk status. The following quote is an example of the nature of debate that participants engaged in.

This is in response to a few of the posters stating that Smoking, Lack of Exersize, Diet are the biggest risk-factors for a heart attack. That Statement is only partially true. While Smoking, Exersize and Diet ARE risk-factors for a heart attack, the BIGGEST risk factor for heart attack is hereditary (Family History). I Work as an EMT and in the Transitional Care Unit (Cardiac Unit) of the hospital and probably half of the people we see in for heart attack dont smoke, exersize and have a fairly good diet, BUT have a family history of heart attack. - alot of them say "Why is this happening to me? I Ate right, exersized, did'nt smoke"...<#7 NON-MED>

Regardless of the specific factors that cause an individual to be at risk, in general, participants seemed to agree that if an individual was deemed to be 'at risk', then a device of some sort would be useful. In many cases an AED was considered to be a sensible investment, particularly for those living in rural locations.

An AED is a wonderful item to have if you or a loved-one have an existing heart condition or significant family history of such (talk to your health provider), especially if you live in a medically underserved area. <#72 NON-MED>

## Discussion

This study explored the parameters of public opinions around home AEDs, using consumer opinions posted on the Internet. The results of a thematic analysis revealed five high-level themes: usability, usefulness, cost, emotional implications, and risk status. Usability functions allowed caregivers to substantiate their belief that the device possessed relevant intelligence thus justifying the notion that the device itself was responsible for the outcome of a SCA intervention. In terms of usefulness, the most common view held by those that did not claim any medical expertise, was that the home AED is a crucial piece of equipment to have within the home, and makes the difference between life and death. Those claiming some level of expertise, however, believed that it was necessary to undertake some level of preparatory training to ensure the swift and effective deployment of the device in the event of a SCA event. Cost issues were variously framed against a backdrop of the inestimable value of a saved life or more conventional weighing of costs and benefits. Home AEDs were also considered to provide significant unquantifiable benefits which more than justified the cost. Emotional implications such as anxiety and regret were anticipated for those that opted not to own a device, and conversely, joy and peace of mind were seen as the positive effect of owning a device. Further arguments in favour of AEDs were developed in relation to risk status, i.e. it was seen to be a wise purchase for those with a family history of heart problems. For those with the lack of such a history, both altruism and a construction SCA events occurring randomly and without warning, were used to claim that AED was a sensible purchase.

### Strengths and Limitations

Internet-based consumer opinion data is readily available within the public domain, and has provided a comparatively rapid and inexpensive primary data source for this study, compared with more traditional data collection methods such as face to face interviews and focus groups, for eliciting participant perspectives on health issues. A number of studies in the health care research domain have begun to explore the use of online discussion groups in general [18,19,62,63]. The work of Nelson [32] provides a precedent for the use of online consumer opinion forums. However, to the best of our knowledge, no research has used online consumer opinion forums to explore health claims located around a particular product. The material analysed in this study suggests that the consumer opinions contain rich qualitative material around the usability and usefulness of the product itself but importantly for present purposes they provide access to detailed reasoning relating to constructions of cost, prevention, the role of affect and of susceptibility.

Consumer opinion forums may be particularly useful in providing data for emerging technologies which have not yet achieved high levels of home market uptake, such as home AEDs. Such data provides timely insights into the content of developing consumer opinion that may otherwise be difficult to access, especially if there is an interest in capturing the views of those that have strong opinions at the early stage of the innovation being adopted. Such information should also prove useful for those charged with the task of communicating around new and emerging health care technologies. It might be a valuable insight in this instance to note, for example, the emotional needs that the AED met and the way in which they rendered the metrics of cost-benefit to be largely irrelevant.

We recognise of course that there are a range of legitimate concerns around the fact that participant characteristics are largely unknown. Although it is a truism to say that qualitative work is not primarily concerned with representativeness, the lack of ability to explore the links between socio-demographic and other personal characteristics and the views expressed is certainly not ideal. Additionally we do not know how the views of those who post AED reviews compare with those have bought an AED and do not post a review, or indeed to what extent, if any, the views of those that contribute to consumer opinions fora represent the views of those that do not. There may also be questions about how the conclusions drawn from an analysis of online consumer opinion forums on a particular topic compares with those derived from more traditional methods such as face to face interviews or focus groups. With this in mind, we can briefly reflect on the key findings of this study in the context of existing research.

### Comparison with existing research

A study by Chen, Eisenberg and Meischke [44], interviewed 31 participants to investigate positive and negative attitudes towards home AEDs. All participants were provided with a home AED unit and received training in its use prior to interviews. There was some similarity between the research questions of this study and our own. For example, it explored the perceived risk of SCA and perceived likelihood of successful defibrillation via an AED, and the perceived positive and negative attitudes towards owning a home AED. Obviously these questions were addressed with a pre-designed data collection protocol. Perhaps surprisingly, there was clear similarity between the two studies in many of the themes and insights that emerged. Using the themes of this study as a baseline, Table 2 provides an indication of where these were echoed by Chen, Eisenberg and Meischke's analysis.

**Table 2:** Comparison of findings with Chen, Eisenberg and Meischke [44]

	Consumer Opinion Forum Themes	**Chen, Eisenberg & Meischke **[44]
Usability	Specific core usability features	
	
	Specific peripheral usability features	
	
	Intelligent device requires minimal input	✓
	
	Device responsible for outcome	✓

Usefulness	Device will save lives	✓
	
	Early defibrillation critical	✓
	
	Additional training necessary	✓
	
	Invest in alternative resources, not AEDs	
	
	CPR alone is sufficient	

Cost	Cost, small price to pay given the benefits	✓
	
	In public health terms - not effective	
	
	Sense of security justifies cost	
	
	Hope never have to use it	✓
	
	Appropriate to balance cost against risk	✓

Emotional implications	Regret of not purchasing	
	
	Improved quality of life	✓
	
	Peace of mind	✓
	
	Denial of emotional consequences	✓

Risk status	Could happen to anyone at any time	
	
	Family history over-riding factor	✓
	
	ICDs potentially preferable given risk status	✓

There were noteworthy similarities between the two studies. In terms of Usability, the interview study confirmed the higher level findings that the AED device is perceived to be intelligently responsive, which allows the user to take minimal responsibility for the outcome of an intervention. Both studies found that the device was often seen as a 'life-saver' and that time criticality of defibrillation was paramount. The results of the current study thus seem promising particularly when considering the minimal resources required to collect this Internet sourced data, and compared with the resources required by an interview study.

We do not wish to try to attach an unwarranted level of significance to the similarities nor to speculate for possible reasons for the differences - and of course we have not attended to the themes that Chen and Eisenberg found that the consumer forum data did not. However, it is not unreasonable to suggest that when there is a need to gain an initial impression of peoples' views and reasoning relating to an event or a technology, and time or money constraints do not permit more traditional data collection methods, that careful analysis of material contributed via Internet forums may be helpful. The analysis of the present study clearly indicated how arguments about the risks and benefits of AEDs were anchored to other claims, both around health, and around the day to day emotional needs that technologies have to meet if they are to be successful [64]. Communications and recommendations on the part of health professionals would benefit from being aware of these 'hazard templates' [65], and mental models [66], when communicating with patients. The value of such an analysis is arguably further heightened when insights are needed around the views of those that are early adopters and may be hard to identify through conventional recruitment methods.

### Comparison with TAM

In terms of noting the high-level themes identified in this study and exploring the extent to which these findings resonate and differ from of established technology acceptance theory, such as the Technology Acceptance Model (TAM) [48] and the key technology acceptance factors; Perceived Usefulness (PU) and Perceived Ease of Use (PEOU). It seems evident that the Usefulness theme is closely aligned with PU, and likewise Usability is closely aligned with PEOU. In terms of Cost, some factors such as it being a 'small price to pay, given the benefits' and the device providing an added 'sense of security' may also be seen as factors that users considered relating to PU, as were 'peace of mind' and 'improved quality of life' for Emotional factors. The observation that these high-level themes appear to have clear foundations in well established technology acceptance theory, provide further support for the idea that data presented within online consumer opinion forums may provide useful, if partial, representations of public opinions towards AEDs, and perhaps more generally, towards evaluating acceptance, adoption and use of emerging health technologies.

There was however an emotional facet to device acceptance and use that does not seem to be clearly accounted for within the PEOU and PU factors. For example, the tension that was felt between the unquestionable motivation to purchase the device and place it within the home, set against the hope that that the device would never actually be used. This uncovers a perhaps somewhat more complex picture of the relationship that exists between the acceptance of this technology and the Behavioural Intention (BI) to actually use it. This is not surprising, taking into account the emotional implications and perceived level of responsibility an individual may associate with the use of the device and the potentially fatal outcome that attempting to use such a device may be associated with. These finding perhaps offer some explanation of Bardy et al's [34] finding that individuals failed to deploy the AED in the event of witnessing an SCA, despite having the device to hand and having received comprehensive device training. Furthermore, there appeared to be a strong element of moral obligation to accept this product, which appeared to be disassociated from the definitions provided for PU and PEOU. The TAM factors PU and PEOU, currently appear to focus on the operational use of a given technology, and for the former the 'extent to which the technology aids them in performing a task' and the latter 'the extent to which using the technology will be free of effort'. Therefore, there does not appear to be appropriate recognition within TAM of the extent to which moral considerations may play a role in the purchase of a device, or the extent to which emotional considerations may subsequently moderate the relationship between behavioural intention (BI) and actual use (AU), particularly in the context of an emergency situation. The notion that new TAM variables may exist relating to user emotion, is supported by more recent research suggesting that psychological aspects such as emotion should be recognised as influential variables in their own right, which are likely to moderate the link between BI and AU [47]. In light of these findings, there is a need to carry out further systematic research to establish the factors that affect acceptance, adoption and use that are specific to AEDs.

Employing qualitative methods to explore the applicability of TAM has also been shown to be a valuable approach to TAM research. Moreover, online consumer opinion forum data has been shown to serve as novel and valuable source of Web-based data, uncovering multifaceted representations of attitudes and beliefs that enrich the understanding of the applicability of TAM relating to AED technologies. Given the readily available, and naturally occurring, nature of this primary data source, coupled with the fact that it can be rapidly accessed at minimal cost compared with more traditional data collection methods, this study serves as a valuable point of reference against which future TAM related research may exploit the potential of Web-based data sources.

## Conclusions

The findings of this study mapped out the parameters of public debate relating to home AEDs. In particular, this study explored opinions originating from a range of individuals, including device owners, potential device owners and individuals that claimed or did not claim some level of medical expertise. This is a departure from previous home AED studies, which typically explored the opinions of individuals that were provided with a device and training as part of a study [44]. As a result, a number of novel factors were identified that have previously remained undiscovered, including specific core and peripheral usability factors, beliefs that CPR alone may be sufficient treatment for SCA events and opinions relating to the trade-off between device functionality and cost. These findings were also considered in the light of an existing and well established technology acceptance theory TAM. Whilst there appears to be some alignment with PU and PEU, the specific function of the device within an emergency health care setting appears to have an impact on the factors that affect technology acceptance and use. In particular, the tension between the perceived moral obligation of accepting and adopting the device set against the responsibility and emotional implications of actually using the device indicate the importance of considering such factors as having significant influence in their own right. Health care providers and policy makers should be mindful of these public opinions when considering the appropriateness of such devices being placed in the homes of patients. Furthermore, mindfulness of these opinions when disseminating information to members of the public on this topic will result in improved and better informed uptake of this technology where appropriate. Although questions may still remain regarding the extent to which the opinions identified in this study truly represent the full extent of public opinion, these opinions provide a valuable and informed starting point for future more focused studies on the topic that may probe on specific factors identified here.

Consequently, we believe that the opinions expressed by reviewers of such devices have provided valuable and timely insights into the public's understanding of, and attitudes towards AEDs within the home. Furthermore, these opinions provide a point of reference for understanding the user practices and issues that members of the public are expected to consider (dictated by the issues raised in public discussion of this subject) when contemplating the acceptance, adoption and use of home AEDs.

## Competing interests

The authors declare that they have no competing interests.

## Authors' contributions

All authors contributed to the conceptual design of this study. AGM carried out primary data collection. AGM and JB carried out the data analysis. All authors contributed to drafting the manuscript. All authors read and approved the final manuscript.

## Pre-publication history

The pre-publication history for this paper can be accessed here:

http://www.biomedcentral.com/1471-2458/11/332/prepub
